# Senotherapeutics: a possible mitigator of age-related pulmonary disease

**DOI:** 10.1016/j.ebiom.2023.104837

**Published:** 2023-10-11

**Authors:** eBioMedicine

Lung disease is a leading cause of death globally. Older individuals are disproportionally affected by lung disease and, with WHO estimates indicating a worldwide increase in the proportion of people aged 60 years or older from 12% in 2015 to 22% in 2050, morbidity and mortality from pulmonary conditions will inevitably increase in upcoming years. Nevertheless, elucidation of the fundamental mechanisms of ageing might be the basis for different therapeutic approaches and provide new strategies for the treatment of older people with lung disease.

Since Hayflick and Moorhead observed that human fibroblasts can only be maintained in culture for a short time, tissue ageing has become associated with the accumulation of senescent cells, which typically display a senescence-associated secretory phenotype (SASP). The SASP—characterised by the increased secretion of cytokines, chemokines, proteases, and growth factors—is crucial to the establishment of a chronic, low-grade inflammatory state known as inflammageing, which is thought to underlie the development of multiple age-related disorders. An improved understanding of cellular ageing has also increased the intensity of the quest for drugs that can mitigate the effects of time. Since 2015, when Zhu and colleagues published the first report on the ability of dasatinib (an SRC–tyrosine-kinase inhibitor) and quercetin (a natural compound that inhibits the phosphoinositide 3-kinase and other kinases) to eliminate senescent cells in *Aging Cell*, research on therapeutic strategies that target cellular senescence has flourished. Currently available senotherapeutic strategies include senolytics, which selectively kill senescent cells, and senomorphics, which prevent the detrimental effects of senescent cells through SASP suppression.

Idiopathic pulmonary fibrosis (IPF) is a progressive, debilitating, fibrotic lung disease with poor prognosis that mostly affects older people. The discovery that senescent cells and the SASP contribute to IPF-associated inflammation and functional decline led to the first trials in humans that aimed to assess the feasibility of a senolytic intervention. In a two-centre, single-arm, pilot study by Justice and colleagues that was published in 2019 in *eBioMedicine*, the intermittent administration of dasatinib plus quercetin for 3 weeks in 14 people with mild to severe but stable IPF was associated with statistically significant improvements in physical function (ie, 6-min walk distance, 4 m gait speed, and chair-stands time), which was assessed 5 days after the last dose. In this study, senolytic agents were generally well tolerated and no notable adverse changes in clinical biochemistry were found; these early results encouraged further investigations in people with IPF. In a trial published in 2023 in *eBioMedicine*, 12 participants were randomly assigned to receive 3 weeks of either intermittent dasatinib plus quercetin or placebo. The authors found no serious adverse events related to the senolytic intervention, although the placebo group reported fewer overall non-serious adverse events than the dasatinib plus quercetin group. Furthermore, sleep disturbances and anxiety were disproportionately found in the dasatinib plus quercetin group. However, the intermittent senolytic intervention was not associated with statistically significant changes in frailty, pulmonary, or physical function, but the study had been powered to assess feasibility and tolerability.

Older age is also associated with an increased risk of severe COVID-19, so an improved understanding of the contribution of cellular senescence in this context is crucial. In a study in golden hamsters by Delval and colleagues that was published in *Nature Aging* in 2023, aged animal models of SARS-CoV-2 exhibited a greater viral load during the acute phase of infection and an higher level of sequelae during the post-acute phase than young animals. Early treatment with navitoclax, a senolytic drug that acts through the inhibition of BCL-2, reduced the pulmonary viral load in aged animals and improved both early and late lung disease, suggesting that age-associated, pre-existing senescent cells are likely to contribute to COVID-19 severity. Two phase 2, randomised, double-blind, placebo-controlled clinical trials for COVID-19 are currently ongoing with fisetin—a flavonoid with greater senotherapeutic activity in cultured cells than quercetin, according to a study published in *eBioMedicine* in 2018.

In a 2023 Review published in *eBioMedicine*, Wan and colleagues discussed how the accumulation of senescent cells might make the airway more susceptible to environmental challenges, as well as how different senescent structural and immune cells have been associated with major clinical endotypes of people with asthma. The particularities of asthma among older people, including physiology, clinical presentation, and management, suggest that the association between cellular senescence and airway inflammation should be further explored in this population. An improved understanding of senescence-related alterations in asthma might lead to the development of new therapeutic strategies for people with asthma that is not well controlled with standard therapies.

As enthusiasm for potential therapeutic applications increases, we should acknowledge that senotherapeutics are still very new and that their implementation in clinical practice will experience several challenges in upcoming years, as discussed in a Review by Raffaele and Vinciguerra published in *The Lancet Healthy Longevity* in 2022. Whereas senescent-cell accumulation contributes to various age-related conditions, senescence has been associated with fundamental physiological mechanisms, such as tissue repair and tumour suppression. Therefore, caution is warranted regarding potential side-effects. Moreover, although several clinical trials are ongoing, standard protocols for timing, dose, route of administration, and person monitoring are yet to be established. In this context, urinary α-Klotho, a geroprotective protein, has promise. In a study published in *eBioMedicine*, Zhu and colleagues showed that senescence was associated with reduced α-Klotho, partly by increasing the production of activin A and IL-1α. Treatment with dasatinib plus quercetin increased α-Klotho in the urine of people with IPF, suggesting that it could be a useful way to assess disease progression and person response to senolytics.

With ageing populations, research into therapeutic interventions that increase the number of years that people can live in reasonably good health is of the utmost importance. Age-related conditions have been traditionally managed as diseases of distinct causes, but senotherapeutics are a change in this framework; the ageing process, and its multiple facets, is the main target. Exploring new interventions for conditions such as IPF, which substantially impairs the quality of life of people and exhibits a modest response to approved drugs, will probably contribute to the healthy ageing of populations. Large, well conducted clinical trials that provide a comprehensive overview of the safety and adverse-effect profile of senotherapeutics will be crucial to understand their full potential in the management of pulmonary disease. At *eBioMedicine*, we welcome research that contributes to advances in the field of senescence-targeting therapeutic interventions.

